# Low Expression of LDHB Correlates With Unfavorable Survival in Hepatocellular Carcinoma: Strobe-Compliant Article

**DOI:** 10.1097/MD.0000000000001583

**Published:** 2015-10-02

**Authors:** Ruohua Chen, Xiang Zhou, Zhenhai Yu, Jianjun Liu, Gang Huang

**Affiliations:** From the Department of Nuclear Medicine, Ren Ji Hospital, School of Medicine, Shanghai Jiao Tong University, Shanghai, China (CRH, ZX, LJJ, HG); School of Biomedical Engineering, Shanghai Jiao Tong University (YZH); and Department of Cancer Metabolism, Institute of Health Sciences, Chinese Academy of Sciences and Shanghai Jiao Tong University School Medicine, Shanghai, China (HG).

## Abstract

Lactate dehydrogenase B (LDHB) is widely expressed in adult somatic tissue and plays important roles in the development of human cancers. However, the association between LDHB expression and the clinicopathological characteristics of hepatocellular carcinoma (HCC) is not well understood. The study was to detect the expression of LDHB in human HCC and investigate the association between its expression and the clinicopathological characteristics of HCC.

Immunohistochemistry (IHC) analysis was performed to characterize the expression of LDHB in HCC and matched noncancerous tissues. Kaplan–Meier survival and Cox regression analyses were employed to evaluate the prognosis of 75 HCC patients.

The results showed that the expression of LDHB in HCC was significantly lower than that in noncancerous tissues. Moreover, the expression level of the LDHB protein in HCC was correlated with pathological grade (*P* = 0.037), vascular invasion (*P* = 0.037), lymph node metastasis (*P* = 0.016), and tumor-node metastasis (TNM) stage (*P* = 0.007). Cox regression analysis further revealed that LDHB expression is an independent prognostic factor for disease-free survival (*P* = 0.045) and overall survival (*P* = 0.019).

These data are the first to indicate that LDHB expression is a valuable prognostic biomarker for HCC and that low LDHB expression suggests unfavorable survival outcomes in HCC patients.

## INTRODUCTION

Hepatocellular carcinoma (HCC) is the fifth most common malignant tumor and causes more than half a million deaths globally each year.^1^ Surgery and interventional therapy are two common strategies for its treatment. However, its rapid progression and high metastasis rate make this fatal disease very difficult to treat. As a result, the overall 5-year survival rate of HCC is only 70%.^2^ HCC is caused by numerous genetic abnormalities, such as gene amplifications and mutations, chromosomal alterations, as well as epigenetic alterations.^3–5^ However, a single-molecule targeting therapy has yet to be discovered. Although alpha-fetoprotein (AFP) is widely used clinically for early diagnosis and the follow-up of patients with HCC,^6^ there are also AFP-negative HCC patients. Thus, the identification of target molecules that control the biological characteristics of HCC and predict early recurrence and survival of HCC is of great importance.

The preference of cancer cells to produce energy by a high rate of glycolysis followed by lactic acid fermentation, even under adequate oxygen, has been called the Warburg effect or aerobic glycolysis.^7,8^ Lactate dehydrogenase (LDH), which catalyzes the interconversion of pyruvate and lactate, is the key glycolytic enzyme.^9^ LDH is a tetrameric enzyme that composes two types of subunits LDHA (muscle-type, M subunit) and LDHB (heart-type, H subunit).^10^ We and others have demonstrated that LDH A (LDHA) that converts pyruvate to lactate is overexpressed in many malignant tumors and acts as an essential role in tumor metabolism,^11–14^ whereas the role of LDHB is less clear. Some studies revealed that LDHB could enhance proliferation in a subset of lung adenocarcinoma and breast cancer.^15–17^ Other studies demonstrated that the expression of LDHB was suppressed in pancreatic cancer, prostate cancer, and gastric cancer.^9,18,19^ However, less definitive evidence has been reported in HCC, and the link between LDHB and HCC development is poorly understood. Thus, further studies which focus on the roles of LDHB in the diagnosis and treatment for HCC are important and of great interest.

In this study, we examined LDHB expression in primary HCC and the relationship between LDHB expression and clinicopathological characteristics. Furthermore, we evaluated the relationship of LDHB expression with the prognostic significance of HCC patients. Our results suggest that LDHB can be considered as a prognostic marker for both disease-free and overall survival (OS) in HCC.

## MATERIALS AND METHODS

### Patients and Tissue Samples

The subjects were 75 patients who received surgical therapy at Shanghai Jiaotong University–affiliated Ren Ji Hospital between 2007 and 2012. The following were eligibility criteria: the diagnosis of HCC was confirmed by pathologic examination of surgical specimens; patients did not receive chemotherapy, radiotherapy, or other local treatments before or after surgical therapy; complete case records, including age, gender, hepatitis B virus (HBV) infection, a-fetoprotein (AFP) status, tumor stage, and histologic differentiation were available; tissue specimens for immunohistochemical staining were available. Finally, 75 patients were evaluated in this study. We assessed the survival information by telephonic follow-up in the nuclear medicine department of our hospital. The follow-up time ranged from 1 to 67 months, with a median time of 26 months. Tumor staging was determined according to the seventh edition classification tumor-node metastasis (TNM) system of the American Joint Committee.^20^ This work was approved by the ethics committee of Shanghai Jiao Tong University affiliated Ren Ji Hospital and all subjects signed a written informed consent form.

### TMA Construction and IHC Analysis

Seventy-five HCC tissues and matched noncancerous tissues were prepared and TMA was produced by Shanghai Zhuoli Biotechnology Co., Ltd (Shanghai, China). Core tissue biopsies (2 mm in diameter) were taken from formalin-fixed paraffin embedded sections and arranged in the new recipient paraffin blocks. The TMA was cut into 4 mm sections and placed on superfrost charged glass microscope slides. Then, sections were deparaffinized in xylene and rehydrated through ethanol. Endogenous peroxidase activity was then blocked by methanol containing 0.3% hydrogen peroxidase for 30 min. After exposure to 10% serum for 10 min, the sections were incubated at 4°C overnight with the primary antibody (anti-LDHB antibody). The sections were then incubated with the second antibody for 20 min. Finally, the sections were counterstained with hematoxylin. LDHB immunostaining was scored by 2 independent pathologists according to staining intensity and distribution. Where discrepancies occurred by 2 readers, the 2 readers reached a consensus. Almost perfect agreement between the 2 pathologists was found for immunostaining detection (k = 0.86). Staining intensity was scored as follows: 0 (negative), 1 (weakly positive), 2 (moderately positive), and 3 (strongly positive). Intensity score was defined as 0, negative; 1, weak; 2, moderate; and 3, strong. The proportion score was defined as 0, negative; 1, <10%; 2, 11% to 50%; 3, 51% to 80%; and 4, >80% positive cells. The product of the intensity and percentage scores gave rise to the final staining score. The degree of LDHB staining was quantified using a 2-level grading system as follows: <6 indicates low expression, while 6 to 12 indicates high expression.

### Statistical Analysis

The relationship between LDHB protein expression and clinicopathologic parameters of HCC was detected by Chi-squared test or Fisher's exact test. Statistical differences between the groups were compared using 1-way analysis of variance (ANOVA) and *t* tests. For survival analysis, Cox proportional hazards regression models, in which the effect of covariates is to multiply the hazard function by a function of the explanatory covariates, have achieved widespread application in the analysis of time-to-event data, with censoring and covariates. In the present study, univariate and multivariate analyses were performed using Cox proportional hazards regression models to identify important factors that statistically associated with disease-free survival (DFS) and OS status. The Kaplan–Meier method was used to analyze the correlation between LDHB expression and the prognosis of HCC patients. For all tests, *P* value <0.05 was considered statistically significant. All statistical analyses were performed using SPSS (version 13.0; SPSS Inc.).

## RESULTS

### Summarization of Clinical Information of 75 HCC Patients

Table 1 summarizes clinicopathological characteristics of 75 HCC patients. This study included 67 men and 8 women. The mean age of the 75 patients was 51 years, ranging from 33 to 76 years. Sixty-three percent of the patients had high AFP level (>20 mg/L) and 73% of the patients were HBV-positive. On the basis of the pathological grade, most of the patients were at grade 2 (66.7%), followed by grade 3 (18.7%) and grade 1 (14.7%). Forty-five patients encountered vascular invasion and lymph node metastasis was detected in 10 patients. Most of the patients were in stage II (41.3%), followed by stage I (26.7%), stage III (18.7%), and stage IV (13.3%).

**TABLE 1:**
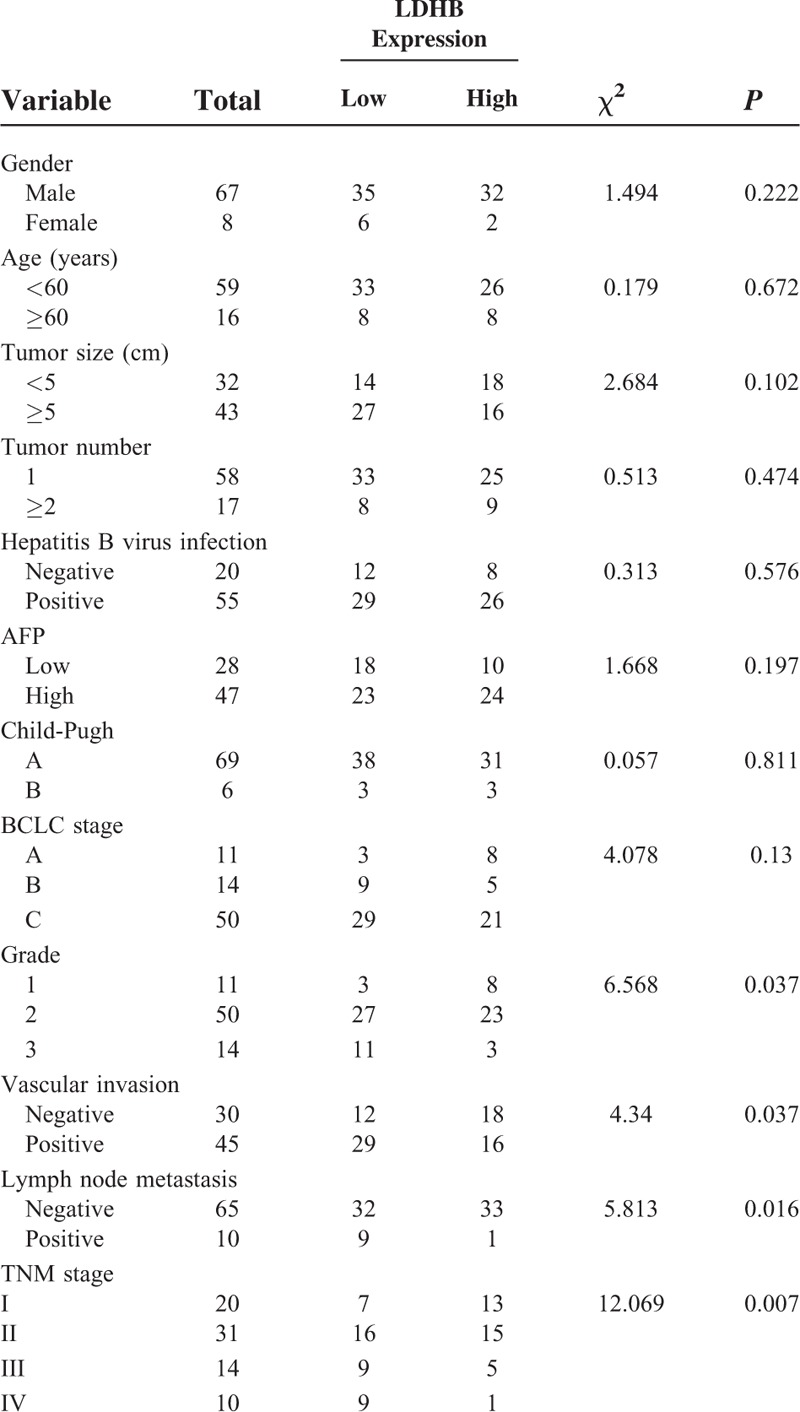
Relationship Between LDHB and Clinicopathological Characteristics in HCC

### LDHB Expression is Downregulated in Primary HCC Compared With Normal Liver Tissue

**FIGURE 1:**
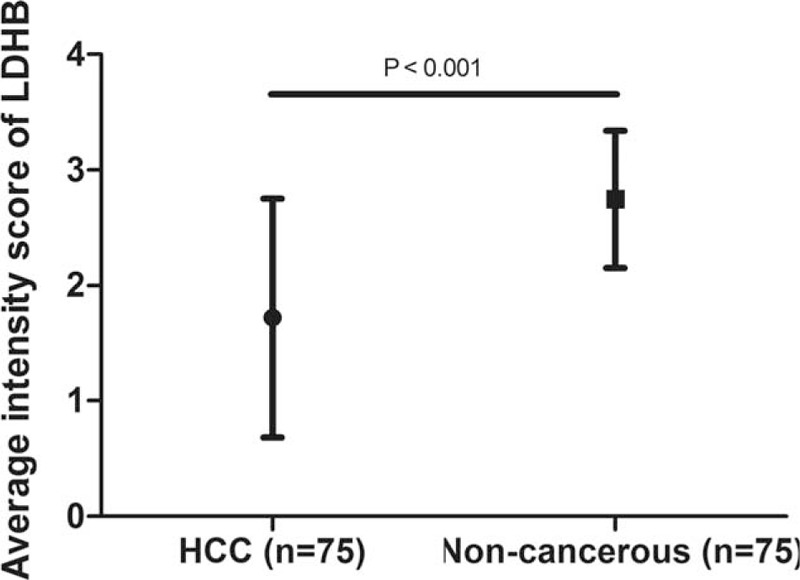
LDHB expression in hepatocellular carcinoma (HCC) tissues and tumor adjacent noncancerous tissues. Immunohistochemistry (IHC) demonstrated that the mean intensity score of LDHB in HCC tissues (1.720 ± 1.034) was significantly lower than that of in matched noncancerous tissues (2.747 ± 0.595).

**FIGURE 2:**
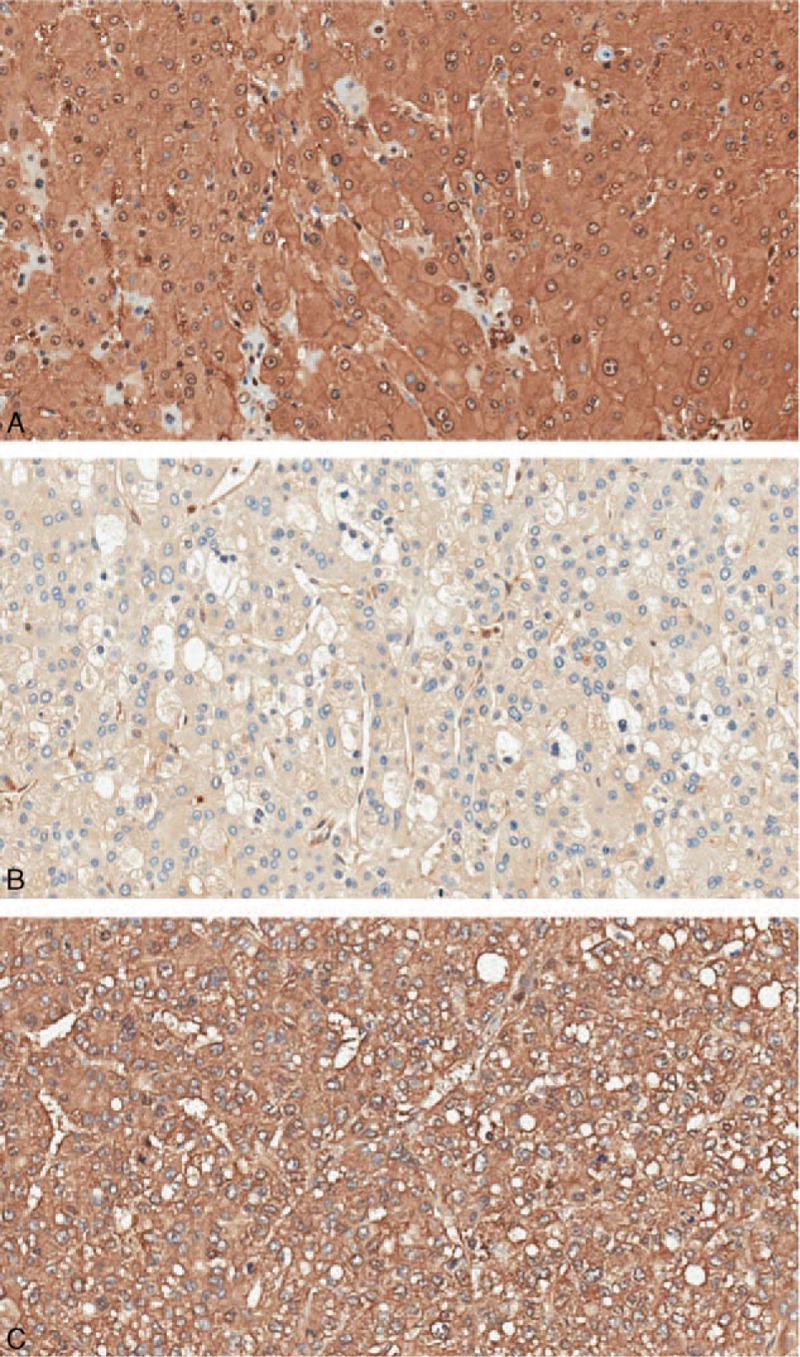
Representative pattern of LDHB protein expression in HCC and corresponding noncancerous tissues (200×). (A) Nonmalignant liver tissues showing strong LDHB staining. (B) HCC tissue showing low expression of LDHB. (C) HCC tissue showing strong LDHB staining.

We compared the expression of LDHB protein between 75 matched normal/cancer specimens from the same patients. High LDHB expression was detected in 64 of 75 (86.3%) normal tissue samples and only 34 cases of 75 HCC tissue samples (45.3%) exhibited high LDHB expression. As is shown in Figure 1, the mean intensity score of LDHB in HCC tissues (1.720 ± 1.034) was significantly lower than that of in corresponding noncancerous tissues (2.747 ± 0.595). LDHB staining was localized predominantly in the cytoplasm and the representative LDHB staining in HCC is shown in Figure 2.

### The Relationships Between LDHB Expression and the Clinicopathological Characteristics

We further analyzed the correlation between LDHB expression and the clinicopathological characteristics of HCC patients. As summarized in Table 1, there was no significant difference between LDHB expression and gender, age, tumor size, tumor number, AFP status, HBV infection, Child-Pugh, and BCLC stage. However, LDHB expression was markedly associated with pathological grade (*P* = 0.037), vascular invasion (*P* = 0.037), lymph node metastasis (*P* = 0.016), and TNM stage (*P* = 0.007).

### The Prognostic Significance of LDHB Protein in HCC

Univariate analysis showed that LDHB expression (*P* = 0.002), vascular invasion (*P* = 0.025), lymph node metastasis (*P* = 0.001), and TNM stage (*P* = 0.001) were correlated with OS of HCC patients (Table 2). In addition, LDHB expression (*P* = 0.005), lymph node metastasis (*P* < 0.001), and TNM stage (*P* = 0.002) were also associated with the DFS in 75 HCC patients (Table 3). By Cox regression, low LDHB expression (*P* = 0.045) and lymph node metastasis (*P* = 0.04) indicated a poor DFS. Meanwhile, LDHB expression (*P* = 0.019) was also identified as independent prognostic factors for OS (Tables 2 and 3).

**TABLE 2:**
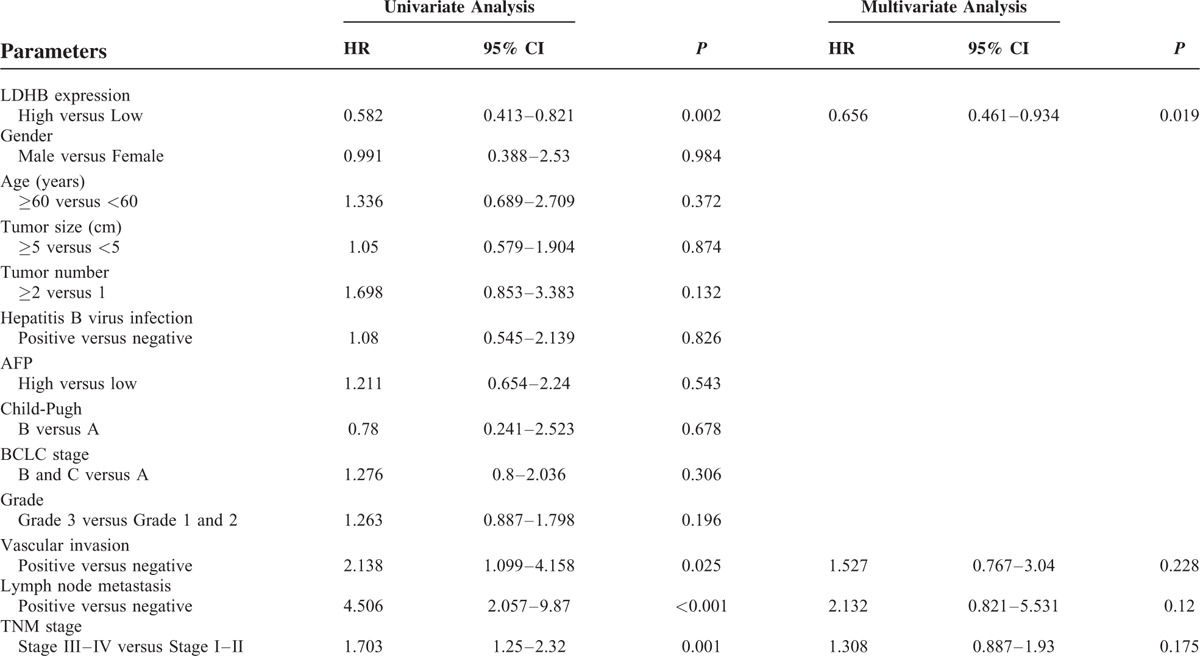
Univariate and Multivariate Analysis of Prognostic Factors in HCC for Overall Survival

**TABLE 3:**
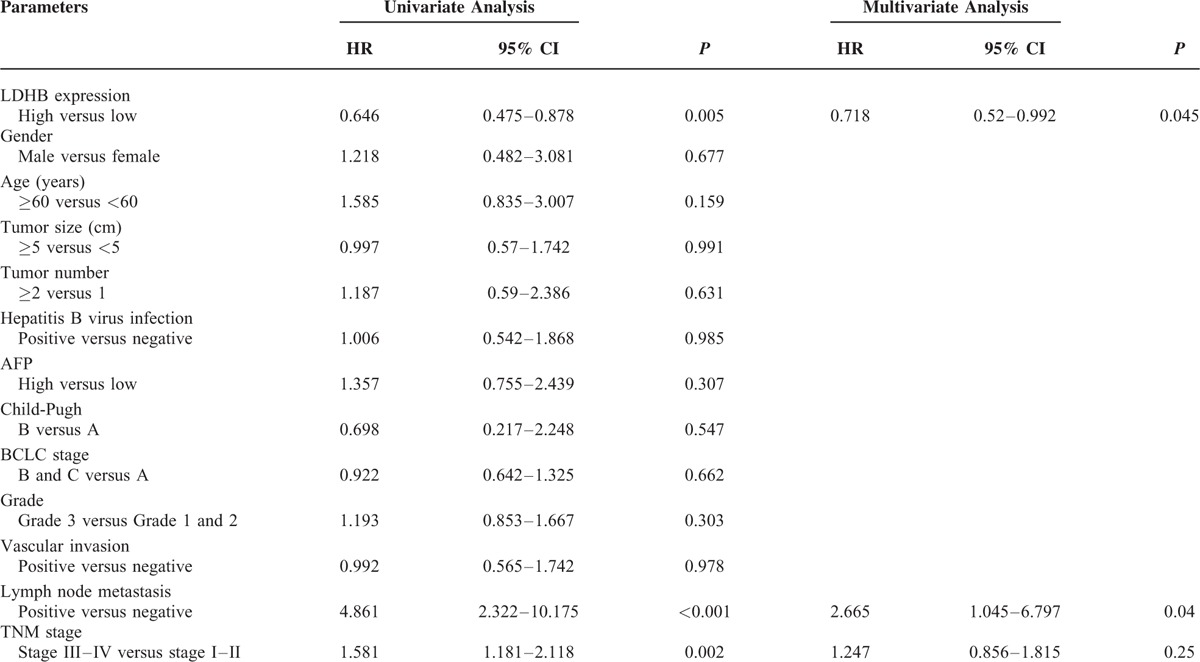
Univariate and Multivariate Analysis of Prognostic Factors in HCC for Disease-Free Survival

**FIGURE 3:**
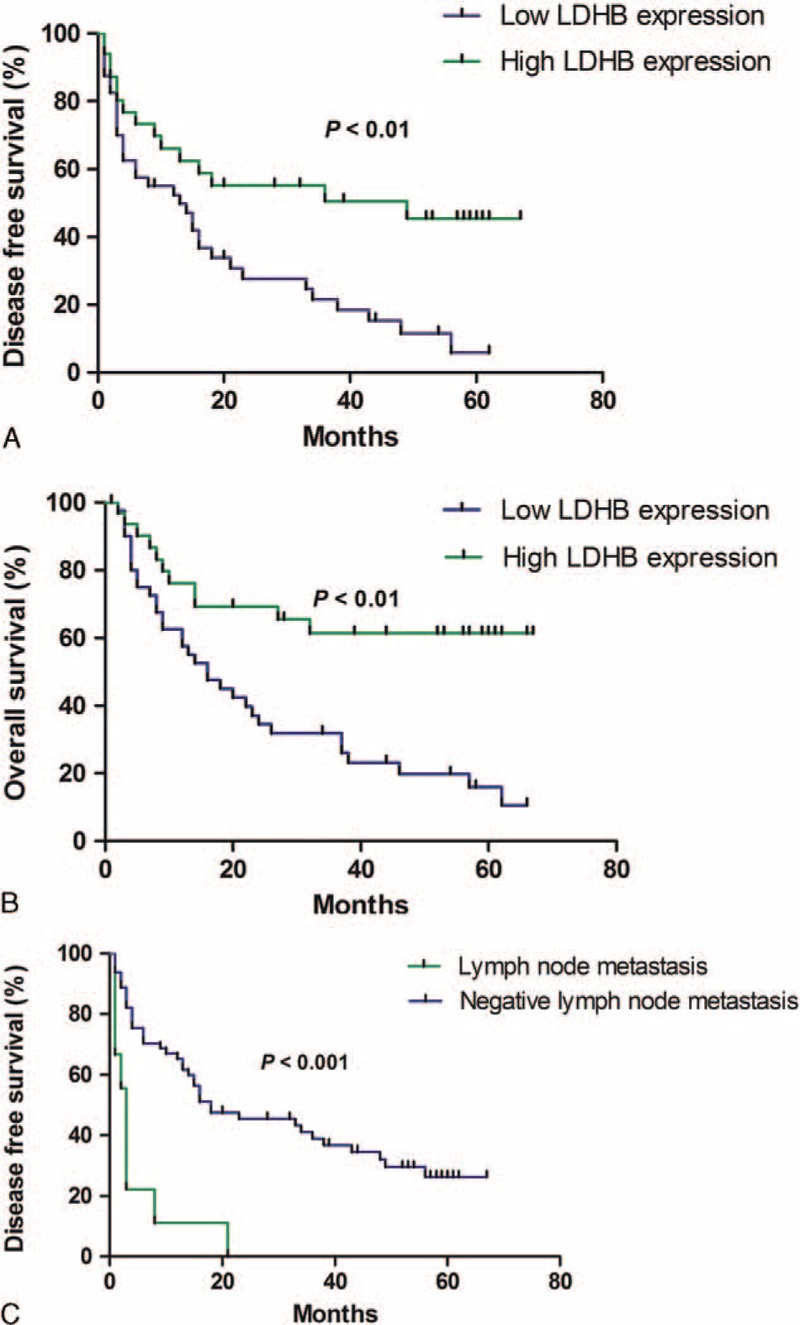
Survival analysis of 75 HCC patients by Kaplan–Meier method. (A) Disease-free survival rate in patients with low LDHB expression was significantly lower than that in patients with high LDHB expression. (B) Overall survival rate in patients with low LDHB expression was significantly lower than that in patients with high LDHB expression. (C) Disease-free survival rate in patients with positive lymph node metastasis was significantly lower than that in patients with negative lymph node metastasis.

The DFS and OS rates for the patients with HCC were 33.3% and 41.3%, respectively. Kaplan–Meier survival curves subsequently demonstrated that HCC patients with low LDHB expression presented a significantly unfavorable DFS time and OS time (both *P* < 0.01). The DFS and OS rates for the patients with low LDHB expression were 14.6% and 19.5%, respectively, whereas the rates were 55.9% and 67.6%, respectively, for the patients with high LDHB expression. In addition, HCC patients with lymph node metastasis experienced poor DFS rate (Figure 3).

## DISCUSSION

Lactate dehydrogenase is a tetrameric enzyme composed of LDHA and LDHB. LDHA is overexpressed in many cancers and plays a crucial role in tumor proliferation, invasion, and metastasis,^11,12,21^ whereas the role of LDHB depends on different types of tumors and remains more elusive. Previous study observed decreased LDHB expression in HCC cells. They also revealed that decreased expression of LDHB enhanced HCC cells invasiveness via mitochondrial defects.^22^ Regardless, the detailed understanding of the function of LDHB in HCC, especially regarding prognostic characteristics, is limited.

Previous findings have indicated that LDHB is suppressed in many kinds of cancer due to promoter hypermethylation, including prostate, gastric, and pancreatic cancer.^9,18,19^ Loss of LDHB in cancer cells led to elimination of LDH1 to 4 and leaved only LDH5 that was expected to catalyze the conversion of pyruvate to lactate.^9^ Meanwhile, it is worth noting that LDHB upregulation has been reported in the context of other tumor types. For example, LDHB is overexpressed and required for the growth of KRAS-dependent lung adenocarcinomas. LDHB expression in lung adenocarcinomas correlates with poor clinical outcome.^15^ In this study, we first investigated LDHB expression in HCC specimens by immunohistochemistry (IHC) analysis. Consistent with previous findings concerning prostate and pancreatic cancer,^9,18^ our results suggested a significantly lower level of LDHB expression in HCC tissue samples than in noncancerous tissue samples. Furthermore, LDHB expression was negatively correlated with pathological grade, vascular invasion, lymph node metastasis, and TNM stage. These data indicated that LDHB were suppressed aberrantly in a significant fraction of HCC and suggested strongly that LDHB might play critical roles during tumor development and serve as novel diagnostic biomarkers for HCC.

For survival analysis, Cox proportional hazards regression models, in which the effect of covariates is to multiply the hazard function by a function of the explanatory covariates, have achieved widespread application in the analysis of time-to-event data, with censoring and covariates. In the present study, a univariate analysis was firstly chosen to detect important factors that may influence the prognosis of HCC patients; a multivariate analysis was then performed to identify the authenticity and validity of the prognostic factors detected. Finally, we screened the prognostic factors LDHB expression and lymph node metastasis for DFS and LDHB expression for OS. The data demonstrated that low LDHB expression was associated with a poor prognosis in patients with HCC. Patients expressing low levels of LDHB exhibited unfavorable outcomes for both DFS and OS. A Kaplan–Meier analysis also verified that HCC patients with low LDHB expression showed a significantly unfavorable lifespan, including DFS and OS.

Although the significance of LDHB in cancers is still inadequate and elusive, several studies have indicated its critical role in tumor development, and LDHB have been identified as potential therapeutic targets for the treatment of malignant tumors.^22^ LDHB can also be used as a metabolic marker of response to neoadjuvant chemotherapy in breast cancer.^17^ Meanwhile, restored expression of LDHB repressed cell proliferation, invasion, and migration in pancreatic cancer.^18^ Thus, demethylating agents might be a promising new therapeutic strategy for prostate and pancreatic cancer. Considering the tumor-suppressed role of LDHB in HCC cell lines^22^ and tissues, the application of restoring expression of LDHB such as demethylating agents may be effective in HCC treatment, which requires further studies.

In summary, we provide evidence that LDHB are suppressed in a significant fraction of HCC. We also conclude for the first time that LDHB can be as a prognostic marker for both DFS and OS in HCC. In addition to be a prognostic marker, our data suggest that overexpressing LDHB may provide a strategy for developing novel therapeutics for HCC. However, the limitation of this study was the small sample size. Further studies that include more clinical samples are needed to unravel the roles of LDHB during the progression and recurrence of HCC.

## References

[1] Parkin DM Bray F Ferlay J Global cancer statistics, 2002. *CA Cancer J Clin* 2005; 55:74–108. 15761078 10.3322/canjclin.55.2.74

[2] Bosch FX Ribes J Diaz M Primary liver cancer: worldwide incidence and trends. *Gastroenterology* 2004; 127:S5–S16. 15508102 10.1053/j.gastro.2004.09.011

[3] Revill K Wang T Lachenmayer A Genome-wide methylation analysis and epigenetic unmasking identify tumor suppressor genes in hepatocellular carcinoma. *Gastroenterology* 2013; 145:1424–1435. 24012984 10.1053/j.gastro.2013.08.055 PMC3892430

[4] Calvisi DF Ladu S Gorden A Mechanistic and prognostic significance of aberrant methylation in the molecular pathogenesis of human hepatocellular carcinoma. *J Clin Invest* 2007; 117:2713–2722. 17717605 10.1172/JCI31457 PMC1950459

[5] Nishida N Goel A Genetic and epigenetic signatures in human hepatocellular carcinoma: a systematic review. *Curr Genomics* 2011; 12:130–137. 21966251 10.2174/138920211795564359 PMC3129047

[6] Marrero JA El-Serag HB Alpha-fetoprotein should be included in the hepatocellular carcinoma surveillance guidelines of the American Association for the Study of Liver Diseases. *Hepatology* 2011; 53:1060–1061. 21374678 10.1002/hep.24033

[7] Warburg O On the origin of cancer cells. *Science* 1956; 123:309–314. 13298683 10.1126/science.123.3191.309

[8] Gatenby RA Gillies RJ Why do cancers have high aerobic glycolysis? *Nat Rev Cancer* 2004; 4:891–899. 15516961 10.1038/nrc1478

[9] Leiblich A Cross SS Catto JW Lactate dehydrogenase-B is silenced by promoter hypermethylation in human prostate cancer. *Oncogene* 2006; 25:2953–2960. 16547507 10.1038/sj.onc.1209262

[10] Drent M Cobben NA Henderson RF Usefulness of lactate dehydrogenase and its isoenzymes as indicators of lung damage or inflammation. *Eur Respir J* 1996; 9:1736–1742. 8866602 10.1183/09031936.96.09081736

[11] Zhou X Chen R Xie W Relationship between 18F-FDG accumulation and lactate dehydrogenase A expression in lung adenocarcinomas. *J Nucl Med* 2014; 55:1766–1771. 25342384 10.2967/jnumed.114.145490

[12] Sheng SL Liu JJ Dai YH Knockdown of lactate dehydrogenase A suppresses tumor growth and metastasis of human hepatocellular carcinoma. *FEBS J* 2012; 279:3898–3910. 22897481 10.1111/j.1742-4658.2012.08748.x

[13] Fantin VR St-Pierre J Leder P Attenuation of LDH-A expression uncovers a link between glycolysis, mitochondrial physiology, and tumor maintenance. *Cancer Cell* 2006; 9:425–434. 16766262 10.1016/j.ccr.2006.04.023

[14] Le A Cooper CR Gouw AM Inhibition of lactate dehydrogenase A induces oxidative stress and inhibits tumor progression. *Proc Natl Acad Sci U S A* 2010; 107:2037–2042. 20133848 10.1073/pnas.0914433107 PMC2836706

[15] McCleland ML Adler AS Deming L Lactate dehydrogenase B is required for the growth of KRAS-dependent lung adenocarcinomas. *Clin Cancer Res* 2013; 19:773–784. 23224736 10.1158/1078-0432.CCR-12-2638

[16] McCleland ML Adler AS Shang Y An integrated genomic screen identifies LDHB as an essential gene for triple-negative breast cancer. *Cancer Res* 2012; 72:5812–5823. 23139210 10.1158/0008-5472.CAN-12-1098

[17] Dennison JB Molina JR Mitra S Lactate dehydrogenase B: a metabolic marker of response to neoadjuvant chemotherapy in breast cancer. *Clin Cancer Res* 2013; 19:3703–3713. 23697991 10.1158/1078-0432.CCR-13-0623 PMC3727144

[18] Cui J Quan M Jiang W Suppressed expression of LDHB promotes pancreatic cancer progression via inducing glycolytic phenotype. *Med Oncol* 2015; 32:589. 10.1007/s12032-015-0589-8 25807933

[19] Maekawa M Taniguchi T Ishikawa J Promoter hypermethylation in cancer silences LDHB, eliminating lactate dehydrogenase isoenzymes 1-4. *Clin Chem* 2003; 49:1518–1520. 12928234 10.1373/49.9.1518

[20] An C Choi GH Lee HS Assessment of preoperative magnetic resonance imaging staging in patients with hepatocellular carcinoma undergoing resection compared with the seventh American Joint Committee on Cancer System. *Invest Radiol* 2012; 47:634–641. 22814590 10.1097/RLI.0b013e3182630e8d

[21] Kim JH Kim EL Lee YK Decreased lactate dehydrogenase B expression enhances claudin 1-mediated hepatoma cell invasiveness via mitochondrial defects. *Exp Cell Res* 2011; 317:1108–1118. 21356207 10.1016/j.yexcr.2011.02.011

[22] Zha X Wang F Wang Y Lactate dehydrogenase B is critical for hyperactive mTOR-mediated tumorigenesis. *Cancer Res* 2011; 71:13–18. 21199794 10.1158/0008-5472.CAN-10-1668

